# Sources of Multidrug Resistance in Patients With Previous Isoniazid-Resistant Tuberculosis Identified Using Whole Genome Sequencing: A Longitudinal Cohort Study

**DOI:** 10.1093/cid/ciaa254

**Published:** 2020-03-13

**Authors:** Vijay Srinivasan, Vu T N Ha, Dao N Vinh, Phan V K Thai, Dang T M Ha, Nguyen H Lan, Hoang T Hai, Timothy M Walker, Do D A Thu, Sarah J Dunstan, Guy E Thwaites, Philip M Ashton, Maxine Caws, Nguyen T T Thuong

**Affiliations:** 1 Oxford University Clinical Research Unit, Ho Chi Minh City, Vietnam; 2 Centre for Tropical Medicine and Global Health, Nuffield Department of Medicine, University of Oxford, Oxford, United Kingdom; 3 Pham Ngoc Thach Hospital for Tuberculosis and Lung Disease, Ho Chi Minh City, Vietnam; 4 Peter Doherty Institute for Infection and Immunity, University of Melbourne, Parkville, Australia; 5 Department of Clinical Sciences, Liverpool School of Tropical Medicine, Liverpool, United Kingdom

**Keywords:** tuberculosis, multidrug resistance, isoniazid resistance, whole genome sequencing, rifampicin resistance

## Abstract

**Background:**

Meta-analysis of patients with isoniazid-resistant tuberculosis (TB) given standard first-line anti-TB treatment indicated an increased risk of multidrug-resistant TB (MDR-TB) emerging (8%), compared to drug-sensitive TB (0.3%). Here we use whole genome sequencing (WGS) to investigate whether treatment of patients with preexisting isoniazid-resistant disease with first-line anti-TB therapy risks selecting for rifampicin resistance, and hence MDR-TB.

**Methods:**

Patients with isoniazid-resistant pulmonary TB were recruited and followed up for 24 months. Drug susceptibility testing was performed by microscopic observation drug susceptibility assay, mycobacterial growth indicator tube, and by WGS on isolates at first presentation and in the case of re-presentation. Where MDR-TB was diagnosed, WGS was used to determine the genomic relatedness between initial and subsequent isolates. De novo emergence of MDR-TB was assumed where the genomic distance was 5 or fewer single-nucleotide polymorphisms (SNPs), whereas reinfection with a different MDR-TB strain was assumed where the distance was 10 or more SNPs.

**Results:**

Two hundred thirty-nine patients with isoniazid-resistant pulmonary TB were recruited. Fourteen (14/239 [5.9%]) patients were diagnosed with a second episode of TB that was multidrug resistant. Six (6/239 [2.5%]) were identified as having evolved MDR-TB de novo and 6 as having been reinfected with a different strain. In 2 cases, the genomic distance was between 5 and 10 SNPs and therefore indeterminate.

**Conclusions:**

In isoniazid-resistant TB, de novo emergence and reinfection of MDR-TB strains equally contributed to MDR development. Early diagnosis and optimal treatment of isoniazid-resistant TB are urgently needed to avert the de novo emergence of MDR-TB during treatment.

## Teaser

We investigated the sources of multidrug-resistant (MDR) tuberculosis (TB) in patients with isoniazid-resistant TB treated with first-line anti-TB therapy and show that reinfection with a new MDR-TB strain was just as common as the emergence of rifampicin resistance among these patients.

Tuberculosis (TB), caused by *Mycobacterium tuberculosis*, kills more people each year than any other single pathogen [1]. Resistance to the first-line anti-TB drug isoniazid is the most common drug-resistant TB, with a global prevalence of 10%, and it is associated with increased risk of treatment failure and emergence of multidrug-resistant (MDR) TB with standard first-line TB therapy (11% and 8%, respectively) compared to drug-susceptible TB (1% and 0.3%, respectively) [2, 3]. Emergence of MDR-TB strains, resistant to both isoniazid and rifampicin, is a major concern with an estimated 600 000 cases of MDR-TB or rifampicin-resistant TB each year [4]. MDR-TB requires longer treatment with more expensive and less effective antibiotics [5]. It is also the precursor for extensively drug-resistant TB [6]. Worldwide prevalence of MDR-TB among patients newly diagnosed with TB is approximately 3.4% compared to 18% among patients diagnosed for a subsequent time [1]. Treatment success remains low at about 56% [1]. Vietnam, where this study is set, has been among the top 20 countries with the highest TB and MDR-TB burden, in absolute numbers [1, 7].

MDR-TB strains isolated from patients initially with susceptible strains have in some past studies been ascribed to reinfection with a MDR-TB strain [8, 9]. However, recent data suggest de novo emergence of MDR-TB may be playing a more significant role than previously thought. An analysis of a global data set of *M. tuberculosis* genomes found that isoniazid resistance typically emerges before rifampicin resistance [10], while a recent meta-analysis concluded that the treatment of patients with isoniazid-resistant disease with standard first-line drugs risks the emergence of MDR-TB [3]. Whole genome sequencing (WGS) can be used to distinguish between de novo emergence and reinfection of MDR-TB and can provide genomic evidence to assess the source of MDR-TB [11–13].

A recently published study from Vietnam explored the bacterial risk factors for treatment failure among patients with isoniazid-resistant TB [2]. However, that study did not explore whether patients who re-presented with MDR-TB had been reinfected with new strains or whether the original TB strain had evolved resistance de novo. Here we used WGS on the longitudinally collected isolates from that study to test the hypothesis that standard first-line treatment of patients with isoniazid-resistant TB risks de novo selection for rifampicin-resistant mutations.

## METHODS

### Ethical Approval

The study was approved by the Oxford University Tropical Research Ethics Committee, United Kingdom (OxTREC 030–07) and the Institutional Research Board of Pham Ngoc Thach Hospital in Ho Chi Minh City, Vietnam. All participants provided written informed consent.

### Patient Recruitment

Between December 2008 and June 2011, newly diagnosed patients with smear-positive pulmonary TB were recruited in Ho Chi Minh City, Vietnam, for a clinical study investigating the bacterial risk factors for treatment failure among patients with isoniazid-resistant TB [2]. Recruitment was restricted to new adult patients (aged ≥ 18 years) without human immunodeficiency virus infection and no prior TB treatment [2]. Initial screening for isoniazid resistance was done using microscopic observation drug susceptibility assay (MODS) [14] with results later confirmed using mycobacterial growth indicator tube (MGIT) [2]. Follow-up was for 24 months with sputum collected, where this could be produced at 0, 1, 2, 5, 8, 12, 18, and 24 months after diagnosis. The patients were treated by directly observed treatment, short course (DOTS) with the then standard first-line regimens according to the Vietnamese Ministry of Health guidelines for susceptible, including isoniazid-resistant, TB: 2 months of isoniazid, rifampicin, pyrazinamide, and ethambutol followed by 6 months of isoniazid and ethambutol or 2 months of isoniazid, rifampicin, pyrazinamide, and streptomycin followed by 6 months of isoniazid and ethambutol or other individualized treatment regimens (Supplementary Table 1) [2, 15].

### Culturing *M. tuberculosis* Isolates and Drug Susceptibility Testing

Sputum samples from the patients were used to culture the *M. tuberculosis* isolates in the Pham Ngoc Thach hospital as per the protocol developed from the national TB control program, Vietnam (Supplementary Methods).

### DNA Extraction and WGS

*Mycobacterium tuberculosis* isolates DNA were extracted using cetyltrimethylammonium bromide method [16]. This genomic DNA was used for library preparation using the Nextera XT kit (Illumina) and 150-bp or 300-bp paired end sequencing using MiSeq V2 or V3 reagent kits (Illumina) on the MiSeq sequencing platform (Illumina).

### WGS Analysis

FASTQ data generated on the Illumina MiSeq machine were mapped against the H_37_Rv reference genome (NC_000962.3) using bwa mem [17], and SNPs were called using GATK (version 3.8–1–0-gf15c1c3ef) in unified genotyper mode [18]. These steps were carried out using the PHEnix pipeline (https://github.com/phe-bioinformatics/PHEnix) and SnapperDB [19]. Maximum likelihood phylogenetic analysis was performed by IQ-TREE version 1.6 [20]. *Mycobacterium tuberculosis* lineages, sublineages, and genotypic antibiotic resistance were identified by Mykrobe predictor TB platform [21].

### Genetic Relatedness Analysis

Genetic relatedness between the *M. tuberculosis* isolates was analyzed by constructing a phylogenetic tree of all the longitudinal isolates with WGS data (n = 368). Phylogenetic location and the SNP distance between the baseline and the MDR-TB isolates emerging in each patient were calculated. From base substitution rate of 0.3–0.5 mutations per genome per year in *M. tuberculosis* isolates, and SNP difference between the longitudinal isolates from our study and from the published literature [11, 12, 22], we used a ≤ 5 SNP difference as a cutoff for the de novo emergence of MDR-TB from the initial isolate and > 10 SNP differences as reinfection with MDR-TB. SNP differences between 5 and 10 were described as indeterminate as it was difficult to differentiate either as de novo emergence or reinfection with another strain.

## RESULTS

### Characteristics of Study Participants

**Figure 1.**
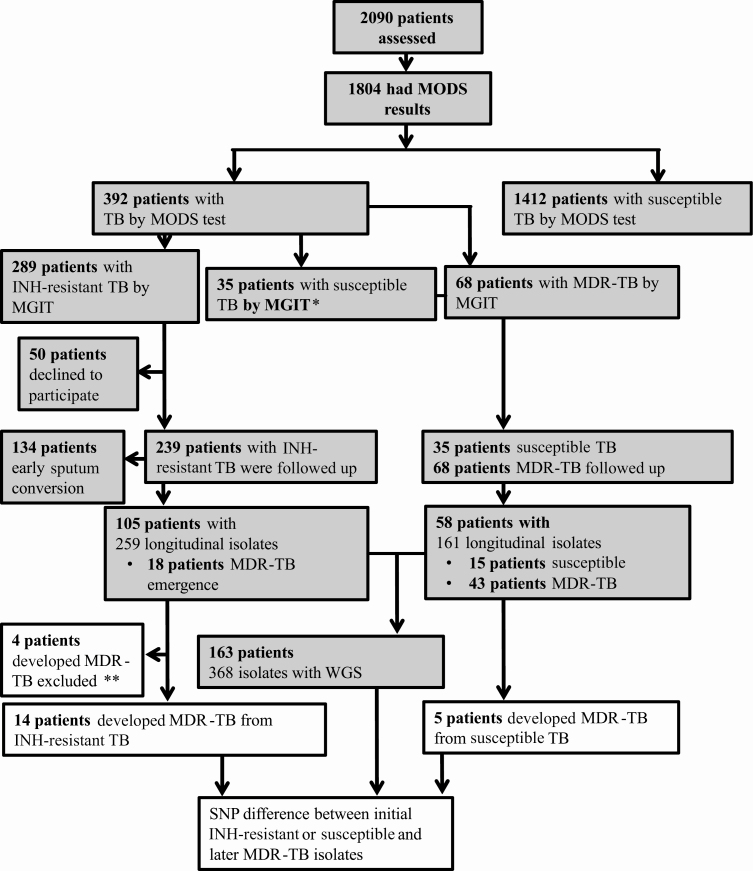
Study flow diagram. Shaded boxes indicate classification of *Mycobacterium tuberculosis* isolates based only on phenotypic drug susceptibility testing (DST); later classification was based on both phenotypic and genotypic DST concordance. *Patients with isoniazid-susceptible tuberculosis (n = 35) by mycobacterial growth indicator tube (MGIT) were followed up due to discordance between microscopic observation drug susceptibility assay (MODS) and MGIT. **Multidrug-resistant tuberculosis excluded due to isolates with known rifampicin resistance mutations in *rpoB* gene but rifampicin-susceptible MGIT results (n =3) and lack of whole genome sequencing (WGS) for further analysis (n = 1). Abbreviations: INH, isoniazid; MDR-TB, multidrug-resistant tuberculosis; MGIT, mycobacterial growth indicator tube; MODS, microscopic observation drug susceptibility assay; SNP, single-nucleotide polymorphism; TB, tuberculosis; WGS, whole genome sequencing.

A total of 2090 consecutively sampled patients were assessed for entry into the study; 1804 patient samples were culture positive and provided TB strains (Figure 1) [2]. Three hundred ninety-two patients had TB strains with isoniazid resistance on MODS; 50 patients declined to be followed up over 24 months and their results were excluded; 68 patients had MDR-TB and 274 had isolates with resistance to isoniazid and susceptibility to rifampicin. Of these 274, confirmatory phenotypic susceptibility testing by MGIT corroborated the isoniazid-resistant result by MODS in 239 cases but reported susceptibility in 35 cases (Figure 1).

Of those patients whose strains were isoniazid-resistant by both MODS and MGIT, 105 of 239 (43.9%) patients produced at least 1 more sputum sample that was culture positive over the 24 months of follow-up, whereas 134 (56.1%) patients had early sputum conversion as their subsequent sputum samples were culture negative. Of those patient strains whose MGIT result was susceptible for isoniazid, despite resistance reported by MODS, 15 of 35 (42.8%) produced subsequent sputum samples that were culture positive and experienced treatment failure, and in the remaining 20 patients subsequent sputum samples were culture-negative. Similarly, only 43 of 68 MDR-TB patients had subsequent sputum samples that were culture positive; for the remaining 25 MDR-TB patients, subsequent *M. tuberculosis* isolates were unavailable. Treatment data for 134 patients with early sputum clearance and 50 patients who declined to participate showed 6 having unfavorable and the rest favorable outcome.

MDR-TB was detected by MGIT during 24 months of follow-up in subsequent isolates from 18 of 105 patients whose baseline isolate was isoniazid resistant by both MODS and MGIT, and 5 of 35 patients with baseline isoniazid-susceptible isolates by MGIT, discordant with MODS result. For 3 of 18 patients with emergence of MDR-TB, the initial isolate was phenotypic rifampicin susceptible by MGIT, but WGS detected rifampicin-resistant mutations and WGS data were lacking for isolates from 1 of the 18 patients. These 4 patients were excluded from analysis (Figure 1). For 163 patients with strains having WGS data, the median age was 41 years, 74.2% were male, and 50.6% reported smoking (Supplementary Table 1).

### Temporal Dynamics of Emergence of MDR-TB in Patients

**Figure 2.**
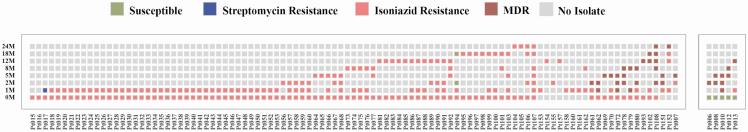
Emergence of multidrug-resistant tuberculosis (MDR-TB) during treatment in patients. Mapping of phenotypic drug susceptibility testing of longitudinal *Mycobacterium tuberculosis* isolates at different months (0M, 1M, 2M, 5M, 8M, 12M, 18M, and 24M) during treatment or recurrence posttreatment from 101 patients initially with isoniazid-resistant TB and 5 patients with susceptible TB. MDR-TB emergence is grouped at the bottom, confirmed based on phenotypic and genotypic drug susceptibility testing (DST). Color code indicates antibiotic susceptibility and no isolate (time points lacking positive *M. tuberculosis* cultures from the patients). Ninety-nine patients initially with isoniazid-resistant TB had DST results for >1 isolate, whereas 2 patients had DST for only initial 0M isolate, as later isolates failed to revive during subculture. Abbreviations: M, month; MDR, multidrug-resistant; Pt, patient.

Of the 14 patients who initially had an isoniazid-resistant strain and developed MDR-TB, 11 did so within the first 5 months of treatment, whereas 3 were diagnosed with MDR-TB 12 or 24 months after completing initial treatment. Of the 5 patients who developed MDR-TB with baseline susceptible strain by MGIT, 4 did so within 5 months of starting treatment and 1 was diagnosed with MDR-TB at 12 months (Figure 2). Among 162 patients, 161 received only 2 or 3 months of rifampicin during the intensive phase, whereas 1 received rifampicin for 6 months during the treatment (Figure 2, Supplementary Table 1).

### Genetic Relatedness Between the Initial and the First MDR-TB Isolate in the Same Patients

**Figure 3.**
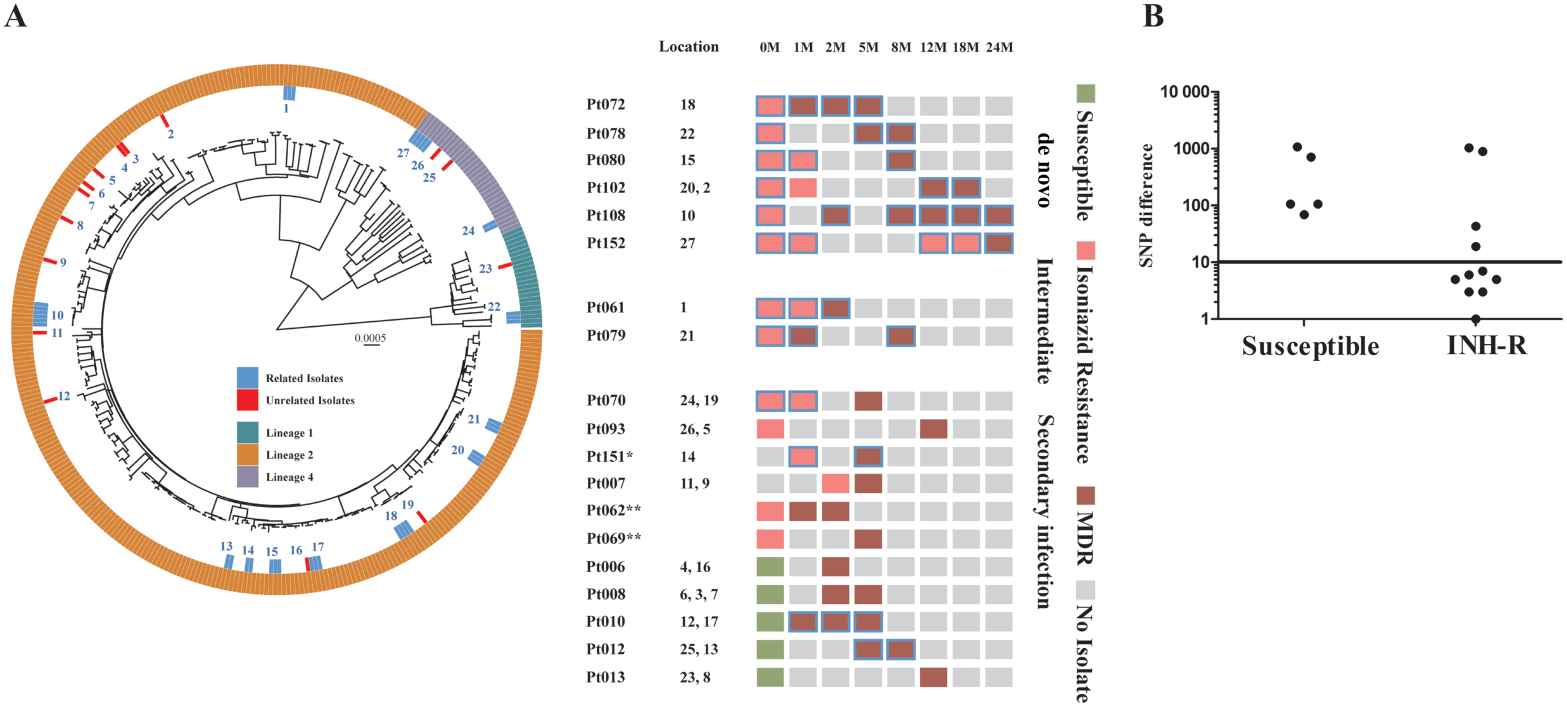
Genetic distance between initial isoniazid-resistant (INH-R) or INH-susceptible isolates and multidrug-resistant tuberculosis (MDR-TB) isolates. *A*, Phylogenetic tree of longitudinal *Mycobacterium tuberculosis* isolates. Emergences of MDR-TB in the phylogenetic tree are indicated in the adjacent panel by patient code, location number in the phylogenetic tree, and collection time points (in months [M]). Patients are grouped based on single-nucleotide polymorphisms (SNPs) difference between initial and MDR-TB isolates; ≥ 5 SNPs (de novo), 6–10 SNPs (intermediate), and >10 SNPs (reinfection) of emergence of MDR-TB from patients initially with INH-R or susceptible TB (color code indicates antibiotic susceptibility). Genetically related isolates from the same patient at different time points are indicated by blue bars in the phylogenetic tree at the respective location number and blue square highlighting the respective collection time points; genetically unrelated isolates at different time points from the same patient are indicated by red bars in the phylogenetic tree at respective location number. Location numbers for isolates from a patient follow the order of collection time point; related isolates from the same patient are given a single location number. Outer ring around the phylogenetic tree indicates different *M. tuberculosis* lineages by color code. *Patient with 19 SNPs difference between initial INH-R and MDR-TB isolates. **Patients with mixed infection removed from phylogenetic tree but analyzed manually. *B*, SNP distance or difference between the initial and the first MDR-TB isolate pair in patients initially with susceptible (SNP range, 69–1077) or isoniazid-resistant isolate (SNP range, 1–1036). One patient had a zero SNP difference between the initial INH-R and MDR-TB isolate, and that data point is not shown in the graph. Black line indicates 10 SNPs cutoff. Abbreviations: INH-R, isoniazid-resistant; M, month; MDR, multidrug-resistant; Pt, patient; SNP, single-nucleotide polymorphism.

To help assess genomic links between isolates and potentially explain MDR-TB acquisition, all longitudinally collected WGS isolates were assessed for genomic relatedness (n = 368 isolates) (Figure 1). In 6 of 14 (43%) patients with initial isoniazid-resistant disease, the subsequent MDR-TB isolates were within 5 SNPs of their original isolates, and not closely related to any other sequenced strains, indicating de novo emergence (Figure 3A and 3B and Supplementary Table 2). One patient appeared to have no SNPs separating the initial isoniazid-resistant and subsequent MDR-TB isolate. However, on closer inspection, a mixed call was detected in *rpoB* at codon 445 with a His to Tyr substitution accounting for 70% of sequencing reads, below the 90% cutoff used for SNP calling (Table 1, patient 080). In 2 cases, the SNP difference between the initial isoniazid-resistant and the MDR-TB isolates was 6 and 7 SNPs, respectively, thus not clearly distinguishing de novo acquisition from reinfection. In the remaining 4 patients, the initial isoniazid-resistant and MDR-TB isolates were separated by 19, 43, 896, and 1036 SNPs, respectively, indicating reinfection (Figure 3A and 3B and Supplementary Table 2), whereas for 2 patients WGS indicated a mixture of strains in their second clinical isolate, with at least 1 of the strains in each mixture being MDR. The initial isoniazid-resistant isolate was not present at the later time-point in either sample. Six of 14 patients were therefore deemed to have been reinfected with MDR-TB (43%).

**Table 1.** Emergence of Genetic Variants in De Novo and Intermediate Emergence of Multidrug-resistant Tuberculosis Isolates

Case ID	Preexisting Antibiotic-Resistant Mutations (WGS)	Preexisting Antibiotic-Resistant Phenotype (MGIT)	Antibiotic-Resistant Mutations Emerged in MDR-TB (Month, % Genetic Variant)	Emerging Antibiotic-Resistant Phenotype (Month)	Other Mutations (Month, % Genetic Variant)	*Mycobacterium tuberculosis* Sublineage	Lineage-Specific SNP	Treatment Regimen^a^
Pt072	*katG S315T* *rpsL K43R* *embB M306I*	INH STR	*rpoB H445Y* (1M, 2M > 90%, 5M = 74%)	RIF (1M, 2M, 5M)		2.2.1.1	*embB (D534D)*	2RHZE/6HE
Pt078	*rpsA* V260I *pncA* C14R	INH STR	*rpoB* H445Y (5M = 66%, 8M > 90%)	RIF (5M, 8M)	*Rv1444c* (M109V): hypothetical protein, (5M = 62%, 8M > 90%) *Rv3806c* (I162L)*, ubiA*^b^ (8M > 90%)	1.1	*Rv3915* (L352L)	2SRHZ/1RHZ/5HE
Pt080	*katG* S315T *rpsL* K88R	INH STR	*rpoB* H445Y (8M = 70%)	RIF (8M)		2.2.1	*Rv0697* (L268L)	2SRHZ/6HE
Pt102	*katG* S315T *rpsL* K43R	INH STR	*rpoB* S450L (1M = 10%, 12M, 18M > 90%)	RIF (12M, 18M)	*Rv2472* (C84R) Hypothetical protein (0M = 73%, 12M, 18M > 90%)	2.2.1.1	*embB* (D534D)	2RHZE/6HE
Pt108	*katG* S315T *rpsL* K43R	INH STR	*rpoB* D435V (2M = 76%, 8M, 12M, 18M, 24M > 90%) *embB* M306V (8M, 12M, 18M, 24M > 90%)	RIF (2M, 8M, 12M, 18M, 24M) EMB (8M, 12M)		2.2.1	*Rv0697* (L268L)	2SRHZ/6HE
Pt152	*fabG1* C-15T *rpsL* K88R	INH STR	*rpoB* D435V (24 M > 90%)	RIF (24M)		4.5	*Rv1524* (P344P)	2SRHZ/6HE
Patients with intermediate SNPs difference								
Pt061	*katG S315T* *embB M306I* *rpsL K43R*	INH STR	*rpoB* S450L (2M = 20%)	RIF (2M)	NADH pyrophosphatase *nudC P239R* (0M = 80%, 2M > 90%)	2.2	*Rv2231c* (A205A)	2SRHZ/6HE
Pt079	*katG S315T* *rpsL K43R*	INH STR	*rpoB H445P* (1M = 77%) *rpoB S450L* (8M > 90%) *embB Q497R* (8M = 88%)	RIF (1M, 8M) EMB (8M)		2.2.1.1	*embB (D534D)*	2SRHZ/6HE

Abbreviations: %, percentage of reads with genetic variant compared to wild-type reference; EMB, ethambutol; ID, identification number; INH, isoniazid; M, month; MDR-TB, multidrug-resistant tuberculosis; MGIT, mycobacterial growth indicator tube; NADH, Nicotinamide adenine dinucleotide (reduced form); RIF, rifampicin; SNP, single-nucleotide polymorphism; STR, streptomycin; WGS, whole genome sequencing.

^a^Treatment regimens: 2RHZE = 2 months of rifampicin, isoniazid, pyrazinamide, and ethambutol; 6HE = 6 months of isoniazid and ethambutol; 2SRHZ = 2 months of streptomycin, rifampicin, isoniazid, and pyrazinamide; 1RHZ = 1 month of rifampicin, isoniazid, and pyrazinamide; 5HE = 5 months of isoniazid and ethambutol.

^b^ *ubiA*—gene involved in *Mycobacterium tuberculosis* cell wall biosynthesis and ethambutol resistance.

Of the 5 patients who initially had susceptible disease and were later diagnosed with MDR-TB, SNP distances between paired isolates ranged from 69 to 1077, indicating reinfection in each instance. Overall, we therefore found that MDR-TB emerged de novo in 6 of 239 (2.5%) patients who were diagnosed with isoniazid-resistant TB by MODS and MGIT, and in 0 of 35 patients whose strains initially tested isoniazid resistant by MODS only (Figure 3A and 3B and Supplementary Table 2).

### De Novo Emergence or Selection of *M. tuberculosis* Variant with Rifampicin-Resistant Mutations During the Emergence of MDR-TB

For 5 of 6 patients with de novo emergence of MDR-TB, mutations known to confer resistance to isoniazid (*katG* S315T in 4/6 cases [66.66%] and *fabG1* C-15T in 1/6 cases [16.66%]) and to streptomycin (*rpsL* K43R and K88R) were detected in the original isolates (Table 1). In the remaining patient, the isoniazid and streptomycin phenotypic resistant isolate had preexisting known pyrazinamide-resistant mutations in the genes *rpsA* and *pncA*, but lacked any known isoniazid- or streptomycin-resistant mutations, so it was probably a resistant phenotype linked to unknown genetic variants. One patient also had an *embB* mutation at the outset, although the ethambutol phenotype was susceptible (patient 072, Table 1). In 155 patients without emergence of MDR-TB, 111 had *katG* S315T (71.61%), 6 had *fabG1* C-15T (3.87%), and the rest lacked any known isoniazid-resistant mutations. There was no significant difference of these mutation frequencies from the strains in which de novo MDR-TB emerged (*P* = .25, Fisher exact test). In each of the 6 de novo MDR-TB cases, known rifampicin-resistant mutations emerged in subsequent isolates (S450L, H445Y, and D435V) (Table 1). The proportion of sequencing reads containing either the relevant *rpoB* mutation or wild-type could be assessed at different time intervals in the 6 patients. One month into treatment, the resistant allele accounted for as few as 10% of reads in 1 patient and for > 90% of reads in another patient’s isolate, although in the former case the phenotype did not convert to “resistant” until the number of resistant alleles had grown further to 90% at 12 months (Table 1). For 4 patients, the resistant *rpoB* allele accounted for between 66% and 76% of reads by 8 months, below the 90% cutoff used for the variant calling, but sufficient to impact the phenotype and be detected by Mykrobe analysis (Table 1). The emergence of an *embB* mutation resulting in resistance to ethambutol could also be observed in 1 case (patient 108) after 8 months of treatment (Table 1). Three other nonsynonymous mutations also emerged, in hypothetical protein *Rv1444c* (M109V) and *Rv3806c*/*ubiA* (I162L) in patient 078 and hypothetical protein Rv2472 (C84R) in patient 102 (Table 1). *ubiA* has previously been linked to ethambutol resistance, although it did not result in a phenotypic change on this occasion [23].

For the 2 patients with intermediate SNP distances between their first and subsequent isolates, known rifampicin-resistant mutations emerged, and in 1 case an ethambutol-resistant mutation also emerged along with a corresponding resistant phenotype (patient 079). Two different rifampicin-resistant variants were observed in patient 079 (Table 1).

Of 8 patients with de novo MDR-TB emergence or an intermediate SNP distance between isolates, 5 patients received 2 months of streptomycin, rifampicin, isoniazid, and pyrazinamide followed by 6 months of isoniazid and ethambutol; 2 patients received 2 months of rifampicin, isoniazid, pyrazinamide, and ethambutol followed by 6 months of isoniazid and ethambutol; and 1 patient received 2 months of streptomycin, rifampicin, isoniazid, and pyrazinamide followed by 1 month of rifampicin, isoniazid, and pyrazinamide followed by 5 months of isoniazid and ethambutol as treatment regimens (Table 1).

For 9 of 11 patients with MDR-TB reinfection but no mixed reads in their MDR-TB isolates, all reinfections were of lineage 2.2.1 with mutation in EsxW-Thr2Ala. This was the same lineage as the initial infection for 5 patients whereas the other 4 were initially infected with strains from lineages 1.1.1.1, 4.8, 4.1.2, and 4.5 (Table 2). The overall prevalence of lineage 2.2.1 among MDR-TB isolates was 79% and 71% among isoniazid-resistant and -susceptible isolates, respectively.

**Table 2.** Sublineages of Initial and Multidrug-resistant Tuberculosis Isolates From Secondary Infection

Case ID	Sublineage of Initial^a^*Mycobacterium tuberculosis* Isolate (Lineage-Specific SNPs)	Sublineage of MDR-TB Isolate (Month)
Pt006	2.2.1 (*Rv0697* [L268L])	2.2.1 (2M)
Pt007	2.2.1 (2M)	2.2.1 (5M)
Pt008	2.2.1	2.2.1 (2M, 5M)
Pt010	2.2.1	2.2.1 (1M, 2M, 5M)
Pt012	4.8 (*Rv3417c* [D51D])	2.2.1 (5M, 8M)
Pt013	1.1.1.1 (*Rv2907c* [V113V])	2.2.1 (12 M)
Pt070	4.1.2 (*Rv0798c* [L172L])	2.2.1 (5M)
Pt093	4.5 (*Rv1524* [P344P])	2.2.1 (12M)
Pt151^b^	2.2.1 (1M)	2.2.1 (5M)

Abbreviations: ID, identification number; M, month; MDR-TB, multidrug-resistant tuberculosis; Pt, patient; SNP, single-nucleotide polymorphism.

^a^“Initial” indicates 0M, 1M, and 2M.

^b^Only 19 SNPs difference between initial and MDR-TB isolate.

There were no instances where rifampicin-resistant alleles were detected in the initial *M. tuberculosis* isolates of either patients who later went on to evolve MDR-TB de novo or due to reinfection at sequencing depth of 30 times.

## DISCUSSION

Here we provide genetic evidence for the de novo emergence of MDR-TB among patients treated with first-line drugs for isoniazid-resistant TB. Contrary to previous studies that found MDR-TB to be the consequence of reinfection [8, 9], de novo emergence of MDR-TB was equally common to reinfection with a separate MDR-TB strain among patients with preexisting isoniazid-resistant TB.

Our findings support the conclusions from recent studies indicating the risk of prior isoniazid resistance in the evolution of rifampicin resistance [3, 10]. Of 239 patients, we observed 6 (2.5%) with initial isoniazid-resistant TB acquiring MDR-TB de novo and 8 (3.3%) who were either reinfected with a new strain that was MDR, or for whom the results were indeterminate. There was no significant difference in clinical presentations between patients with and without emergence of MDR-TB except for drinking alcohol (Supplementary Table 3).

The isolates from patients in Vietnam are not routinely screened for isoniazid resistance [2]. This is also true for patients in many other low- and middle-income countries. Rapid molecular diagnosis methods are available or under development to improve the detection of antibiotic-resistant TB such as Xpert MTB/RIF Ultra for rifampicin resistance and DNA line-probe assays such as the AID TB Resistance LPA and GenoType MTBDRplus VER2.0 for isoniazid and rifampicin resistance detection [24]. It is well understood that suboptimal antibiotic regimens can select for resistant mutations in the *M. tuberculosis* population [25]. All the 6 patients with de novo emergence of MDR-TB as well as the 2 patients with intermediate SNP distances separating their longitudinal isolates were already resistant to streptomycin as well as isoniazid. Two also had mutations conferring resistance to ethambutol leaving rifampicin almost entirely unprotected during the intensive phase, exposing it to selection pressure driving the emergence of rifampicin-resistant variants in the population.

Although treatment regimens for isoniazid-resistant TB have changed to 2 months of rifampicin, isoniazid, pyrazinamide, and ethambutol followed by 4 months of rifampicin, isoniazid, and ethambutol since this study recruited, the emergence of rifampicin resistance during the intensive phase of treatment among our study patients is a major concern. In today’s regimens it is protected only by ethambutol in the continuation phase in patients with isoniazid resistance. Our findings clearly underscore the need for rapid, comprehensive drug susceptibility testing and implementation of new World Health Organization guidelines for treating isoniazid-resistant TB with 6 months of rifampicin, ethambutol, pyrazinamide, and levofloxacin [26].

TB-endemic countries have a higher risk of mixed infection or reinfection [27]. Mixed infection is harder to diagnose, and patients risk being treated with regimens that select for resistant bacterial populations [28]. Reinfection with MDR-TB is a major concern especially where hospitalization, visits to outpatient departments, and attendance to DOTS clinics increase the risk of exposure to other TB patients [29].

Standard culture-based WGS on *M. tuberculosis* isolates cannot rule out the presence of minor resistance alleles prior to treatment [30]. The early detection of emergence of MDR-TB minor variants in the patient can help clinicians to appropriately change the treatment regimen [31].

The Beijing sublineage 2.2.1 was responsible for each patient who was secondarily infected with MDR-TB, consistent with the high prevalence and observation that Beijing sublineage 2.2.1 is involved in enhanced transmission among the host population in Vietnam [32].

There are some limitations to our study. Most importantly, we have only focused on the old 8-month TB treatment regimen that lacks rifampicin in the continuation phase. This was because the strains from a previous study were readily available to us to investigate this important question [2]. This may have decreased the frequency of de novo emergence of MDR-TB from isoniazid-resistant TB, as there was no rifampicin selection pressure after initial 2 months of treatment. However, observing resistance emerge during the intensive phase when rifampicin is supposedly protected by more drugs than in the continuation phase is sobering. MTB/RIF Xpert remains the assay of choice in many low- and middle-income settings but would no more pick up the resistance to ethambutol, pyrazinamide, or second-line injectable drugs now than it would have then. The risks associated with incomplete diagnostics are therefore apparent. A separate weakness is that we cannot rule out the possibility of MDR-TB reinfection with an isolate that is related genetically to the initial isolate, for example from a household contact. We also lacked follow-up data for the patients whose initial MODS screening result was isoniazid susceptible. This may have underestimated the de novo emergence of MDR-TB in patients with a susceptible *M. tuberculosis* isolate.

In conclusion, our study found that de novo emergence of MDR-TB in patients with isoniazid-resistant TB occurred equally frequently to reinfection with MDR-TB in this cohort. It is not routine for drugs other than rifampicin to be screened for resistance at diagnosis. This study provides genetic evidence that such a narrow diagnostic focus risks selection for MDR-TB.

## Supplementary Data

Supplementary materials are available at *Clinical Infectious Diseases* online. Consisting of data provided by the authors to benefit the reader, the posted materials are not copyedited and are the sole responsibility of the authors, so questions or comments should be addressed to the corresponding author.

ciaa254_suppl_Supplementary_Materials

## Notes

***Author contributions.*** S. V., V. T. N. H., D. N. V., and N. T. T. T. designed the study; P. V. K. T., D. T. M. H., and N. H. L. supervised patient recruitment and sample collection; S. V., V. T. N. H., H. T. H., and D. D. A. T. carried out experiments; N. T. T. T., S. V., V. T. N. H., D. N. V., P. M. A., T. M. W., S. J. D., G. E. T., and M. C. analyzed and interpreted the results. All authors contributed to manuscript preparation.

***Acknowledgments*.** The authors acknowledge all participants in the study. The authors also thank the physicians and nurses at the district tuberculosis units, Pham Ngoc Thach Hospital and Oxford University Clinical Research Unit, who contributed to the study.

***Disclaimer.*** The study funding agency did not have any role in study design; data collection, analysis, or interpretation; or writing of the manuscript. The corresponding author had full access to all the data in the study and had final responsibility for the decision to submit the manuscript for publication.

***Financial support*.** This work was supported by the Wellcome Trust (Research Fellowship 081814/Z/06/Z to M. C.); the Wellcome Trust Intermediate Fellowship in Public Health and Tropical Medicine (206724/Z/17/Z to N. T. T. T.); the Wellcome Trust Major Overseas Program (106680/B/14/Z to G. T.) and Wellcome Trust Clinical Research Career Development Fellowship (214560/Z/18/Z to T. M. W.); the Agency for Science, Technology and Research, Biomedical Research Council; and the National Health and Medical Research Council (A*STAR/NHMRC grant APP1056689 to S. J. D.).

***Potential conflicts of interest.*** The authors: No reported conflicts of interest. All authors have submitted the ICMJE Form for Disclosure of Potential Conflicts of Interest.
