# Supplementary material for: Sources of Multidrug Resistance in Patients With Previous Isoniazid-Resistant Tuberculosis Identified Using Whole Genome Sequencing: A Longitudinal Cohort Study
Source: Clin Infect Dis. 2020 Mar 13;71(10):e532–9. doi: 10.1093/cid/ciaa254 (PMC7744982; doi:10.1093/cid/ciaa254)
Supplement: ciaa254_suppl_Supplementary_Materials [file ciaa254_suppl_supplementary_materials.docx]

| Characteristics | n | All patients |
| --- | --- | --- |
| Age (year), median [IQR] | 163 | 41·0 [30·0; 47·0] |
| Gender n (%) | 163 |  |
| Female |  | 42 (25·8%) |
| Male |  | 121 (74·2%) |
| Hepatitis n (%) | 162 |  |
| No |  | 157 (96·9%) |
| Yes |  | 5 (3·09%) |
| Diabetes n (%) | 162 |  |
| No |  | 148 (91·4%) |
| Yes |  | 14 (8·64%) |
| Smoker n (%) | 162 |  |
| No |  | 80 (49·4%) |
| Yes |  | 82 (50·6%) |
| Alcohol n (%) | 162 |  |
| No |  | 97 (59·9%) |
| Yes |  | 65 (40·1%) |
| Regimen (month) n (%) | 162 |  |
| 2RHZE/6HE |  | 113 (69·8%) |
| 2SRHZ/6HE |  | 39 (24·1%) |
| 2SRHZ/1RHZ/5HE |  | 6 (3·70%) |
| 3RHZE/3RH |  | 1 (0·62%) |
| 3RHZE/5HE |  | 3 (1·85%) |

**Supplementary materials**

**Supplementary** **Table 1. Baseline characteristics of patients with isolates having WGS data.**

**Supplementary Table 2. *De novo*, intermediate and reinfection of MDR-TB in patients with susceptible or isoniazid resistant *M. tuberculosis* isolate at enrollment.**

|  | Number of patients with susceptible TB | Number of patients with INH resistant TB |
| --- | --- | --- |
| *De novo* and intermediate MDR-TB emergence | 0 | 6 (43%), and 2 (14%) |
| MDR-TB reinfection | 5 (100%) | 6 (43%) |

|  |  |  |
| --- | --- | --- |

**Supplementary Table 3. Clinical presentation of patients with WGS (n = 163) and *de novo*, intermediate and without emergence of MDR-TB.**

|  |  |  |  |
| --- | --- | --- | --- |
| Characteristics | **Patients without MDR-TB emergence (n=155)** | **Patients with *de novo* and intermediate MDR-TB emergence (n=8)** | **p-value** |
| Age | 41.0 [30.0;47.0] | 41.5 [36.8;43.5] | 0.818 |
| Gender: |  |  | 0.426 |
| Female | 39 (25.2%) | 3 (37.5%) |  |
| Male | 116 (74.8%) | 5 (62.5%) |  |
| Hepatitis: |  |  | 1 |
| No | 150 (96.8%) | 7 (100%) |  |
| Yes | 5 (3.23%) | 0 (0.00%) |  |
| Diabetes: |  |  | 1 |
| No | 141 (91.0%) | 7 (100%) |  |
| Yes | 14 (9.03%) | 0 (0.00%) |  |
| Smoker: |  |  | 0.062 |
| No | 74 (47.7%) | 6 (85.7%) |  |
| Yes | 81 (52.3%) | 1 (14.3%) |  |
| Alcohol: |  |  | 0.042* |
| No | 90 (58.1%) | 7 (100%) |  |
| Yes | 65 (41.9%) | 0 (0.00%) |  |
| Xray_Abnormal: |  |  | 1 |
| No | 16 (10.3%) | 0 (0.00%) |  |
| Yes | 139 (89.7%) | 8 (100%) |  |
| AFB_status: |  |  | 0.545 |
| Mild | 23 (15.0%) | 0 (0.00%) |  |
| Intermediat | 52 (34.0%) | 4 (50.0%) |  |
| Severe | 78 (51.0%) | 4 (50.0%) |  |
|  |  |  |  |

Except for alcohol drinking, p-value (calculated by CompareGroups in R package) was not significant for proportion of distribution of patient’s characteristics between the groups. For hepatitis, Diabetes and smoking, information was only available for 7 patients in emergence of MDR-TB group.

**Supplementary method**

**Culturing *M. tuberculosis* isolates and Drug Susceptibility Testing**

Sputum samples around 3-5 mL were decontaminated by treating with equal volume of 2% sodium hydroxide (NaOH) and 0.5% N-Acetyl-L-Cysteine (NALC) in a 50 mL falcon tube, followed by mixing by vortex for 20 seconds. The treated samples were incubated at room temperature for exactly 15 minutes, and neutralized by adding 35 - 37 mL of H_2_O (ELGA). Samples were centrifuged at 3000 RPM for 15 minutes, supernatant was carefully removed, and the pellet was further centrifuged at 10000 RPM for 15 minutes to remove additional supernatant. The pellet was dissolved in 100 µL of water and used to culture *M. tuberculosis* on LJ tube. Colonies from *M. tuberculosis* cultures from positive sputum samples (n = 448) were scooped for archive and sub-culturing in Mycobacterial Growth Indicator Tube (MGIT) for phenotypic drug susceptibility testing (DST) by BACTEC MGIT 960 SIRE Kit (Becton Dickinson) according to the manufacturer’s protocol in the biosafety level-3 laboratory at the Oxford University Clinical Research Unit. DST was done for streptomycin (1.0 µg/mL), isoniazid (0.1 µg/mL), rifampicin (1.0 µg/mL) and ethambutol (5.0 µg/mL).

**Whole genome sequencing genomic library preparation:**

*M. tuberculosis* genomic DNA was used for library preparation using the Nextera XT kit (Illumina). The libraries were quantified and normalised using KAPA Library Quantification low ROX qPCR mix (Roche) and pooled to 4 nM concentration.

**Whole genome sequence analysis :**  Positions where the majority allele accounted for < 90% of reads mapped at that position, which had a genotype quality of < 30, depth < 5x, or mapping quality < 30 were recorded as Ns in further analyses. Repeat regions of the genome as previously defined [32] were masked and then positions in which at least one strain had SNP passing quality thresholds were extracted and used as the input for IQ-TREE v1·6 [20]. We identified SNPs specific to each *M. tuberculosis* isolate, but not present in other longitudinal *M. tuberculosis* isolates from the same patient, as these isolate specific SNPs could be the causal genetic variant for any phenotypic difference such as antibiotic susceptibility between the longitudinal *M. tuberculosis* isolates. These specific SNPs were identified using bcftools (https://samtools.github.io/bcftools/bcftools.html) isec command e.g. `bcftools isec -p output_name -n -$X sample1.vcf sampleN.vcf` where $X is the number of VCFs being compared minus one. The multiple VCFs produced by this command were then combined with bcftools merge. The inputs VCFs for this process were the filtered VCFs produced by PHEnix. *M. tuberculosis* lineages and sub lineages were identified by Mykrobe predictor TB platform [21] based on the 62 SNPs previously reported for genotyping *M. tuberculosis* isolates [21] and using a genotyping script written in Python 2.5. SNP difference between pairs of *M. tuberculosis* genomes was determined with the Disty program (https://github.com/c2-d2/disty/blob/master/readme.md). We excluded non-tuberculosis mycobacterium (NTM) based on Mykrobe analysis (n = 8). Mixed *M. tuberculosis* isolates were identified based on their shorter root to tip distance as compared to rest of the isolates in the phylogenetic analysis and were excluded (n = 26). For patients, having mixed sample sequenced at MDR emergence time-point (as determined by root to tip distance), the presence of initial isolate was tested for in the mixed MDR emergence time-point (as assessed by searching for the presence of SNPs specific to the initial isolate in the VCF of the mixed sample at low allele frequency).
